# Patterns and determinants of primary care virtual health service use among rural Aboriginal and Torres Strait Islander adults with chronic diseases: a cross-sectional study

**DOI:** 10.1136/bmjopen-2025-115212

**Published:** 2026-05-28

**Authors:** Rezwanul Haque, Annika Luebbe, Matthew R McGrail, Bushra F Nasir, Khorshed Alam, Katharine Wallis, Floyd Leedie, Srinivas Kondalsamy-Chennakesavan

**Affiliations:** 1 Toowoomba Regional Clinical Unit, Medical School, Faculty of Health, Medicine and Behavioural Sciences, The University of Queensland, Toowoomba, Queensland, Australia; 2 Rockhampton Regional Clinical Unit, Medical School, Faculty of Health, Medicine and Behavioural Sciences, The University of Queensland, Rockhampton, Queensland, Australia; 3 School of Business, Law, Humanities and Pathways and Centre for Health Research, University of Southern Queensland, Toowoomba, Queensland, Australia; 4 General Practice Clinical Unit, Medical School, The University of Queensland, Brisbane, Queensland, Australia; 5 Goondir Health Services, Dalby, Queensland, Australia

**Keywords:** Health policy, Health Equity, Australian Aboriginal and Torres Strait Islander Peoples, Primary Health Care, Chronic Disease, eHealth

## Abstract

**Abstract:**

**Objective:**

To examine virtual health service (VHS) device usage patterns and identify factors associated with VHS engagement among Indigenous Australian adults with chronic conditions.

**Design:**

Cross-sectional survey.

**Setting:**

An Aboriginal Community Controlled Health Organisation providing primary healthcare services across rural and remote Queensland, Australia.

**Participants:**

74 consenting Indigenous Australian adults with at least one chronic condition who were registered VHS users.

**Methods:**

Participants completed surveys assessing their use of four Bluetooth-enabled monitoring devices (pulse oximeter, blood glucose monitor, blood pressure monitor, weight scale) over a 2-week recall period. The primary outcome was VHS device usage status, categorised as active versus inactive users. Active users were defined as participants who reported any frequency of device use, while those reporting no use were classified as inactive users. All participants had access to Indigenous health coach support as part of the VHS model. Binary logistic regression was used to identify sociodemographic and geographical factors associated with VHS engagement.

**Results:**

Sixty-four per cent (n=47) were active users, with 73% of these using all four devices concurrently. Among active users, blood pressure monitors showed the highest utilisation (98%), followed by weight scales (91%), blood glucose monitors (89%) and pulse oximeters (86%). Three factors were significantly associated with VHS usage using binary logistic regression: residing in medium rural towns (adjusted OR 4.71, 95% CI 1.23 to 17.94, p=0.02), age 18–65 years (adjusted OR 3.59, 95% CI 1.05 to 12.22, p=0.04) and having multiple chronic conditions (adjusted OR 10.95, 95% CI 1.25 to 95.87, p=0.03) compared with those in more remote areas, aged ≥66 years and with single condition, respectively.

**Conclusion:**

Indigenous-led VHS achieve substantial engagement through culturally grounded health coach support. However, addressing digital connectivity in remote areas, age-appropriate support for older adults and Indigenous workforce development is essential to ensure equitable access and sustained engagement

**STRENGTH AND LIMITATIONS OF THIS STUDY**

- First quantitative study examining virtual health service usage patterns and predictors among Indigenous Australians with chronic conditions in routine Aboriginal Community Controlled Health Organisation primary care.
- Real-world evidence from established practice enhances applicability to similar settings.
- Comprehensive four-device assessment enabled examination of multi-device engagement and geographical disparities.
- Indigenous governance and data sovereignty principles guided culturally appropriate research partnership.
- Cross-sectional design limits ability to establish causal relationships; associations between predictors and engagement can be identified but causation cannot be determined.

## Introduction

 Chronic diseases affect nearly 50% of the Australian population^1^ and continue to rise in prevalence over time,^2^ contributing substantially to the overall burden of disease.^3^ This burden is not distributed equally; the prevalence and severity of chronic diseases are disproportionately higher in rural and remote regions.^3^ In addition, these rural populations face significant structural barriers to accessing timely and appropriate healthcare due to workforce shortages and vast geographical distances, resulting in significantly lower utilisation of essential healthcare services compared with urban centres.^4^

This pervasive health inequity is starkly evident in Aboriginal and Torres Strait Islander Australian communities (hereafter respectfully referred to as *Indigenous Australians*), who experience a 2.3 times higher burden of disease, primarily driven by chronic conditions, compared with their non-Indigenous counterparts.^5^ For Indigenous Australians residing in regional, rural and remote (hereafter collectively referred to as *rural*) communities, these challenges are compounded.^6^ Access to culturally safe and appropriate healthcare is compromised by complex, intersecting barriers, including immense geographical distances, affordability and issues related to cultural relevance, acceptability and approachability.^7^ This combination of barriers results in profoundly poorer quality of life and more severe health outcomes for rural Indigenous populations living with chronic disease.^6^

Digital health, including virtual health services (VHS; remote digital health monitoring using connected devices to transmit patient-generated health data to healthcare providers), is emerging as a crucial component as part of the solution for overcoming the systemic healthcare barriers faced by rural Indigenous patients with chronic disease.^8^ While the global uptake of telehealth was dramatically accelerated by the COVID-19 pandemic,^9 10^ this technological evolution extends beyond crisis management. Contemporary digital health interventions, particularly remote patient monitoring, now provide sophisticated, person-centred platforms capable of real-time data collection, monitoring and feedback and tailored support to drive significant behaviour change and improve health outcomes.^11^ Leveraging these technologies holds immense potential to enhance the effectiveness, equity and responsiveness of health systems for Indigenous people.^12 13^ However, the successful deployment of these tools is contingent on a deeper understanding of their cultural acceptability, safety, relevance and, critically, the factors driving sustained client engagement.^14^

However, the evidence base also highlights that VHS is not a universal solution, and a number of well-documented barriers and clinical risks temper its potential. Low digital literacy represents a significant challenge, particularly among older adults, individuals with lower educational levels, and rural and Indigenous populations, where limited familiarity with technology has been shown to substantially reduce successful engagement.^15 16^ For older populations specifically, age-related sensory impairments, including hearing and vision loss, present additional barriers to effective virtual consultation, with studies reporting that such limitations compromise communication quality and increase the likelihood of clinically important information being overlooked during remote encounters.^17 18^ Structural inequities in digital infrastructure further constrain access, with inadequate broadband, limited device ownership and poor internet connectivity in rural areas constituting persistent barriers to VHS uptake.^19 20^ At the clinical level, virtual care introduces inherent limitations in the capacity to conduct physical examinations, raising concerns about diagnostic accuracy, incomplete assessment and patient safety, particularly for complex or new presentations.^21 22^ Data privacy, cybersecurity and the absence of standardised clinical governance frameworks have also been identified as risks associated with virtual care at scale.^22 23^ Despite these challenges, telehealth infrastructure and policy frameworks have continued to develop in Australia, creating opportunities to examine how these barriers and facilitators shape actual utilisation patterns in community-controlled health settings.

In Australia, VHS such as telehealth have been established for decades, providing healthcare access to rural areas.^24^ Telehealth items were first introduced to its Medical Benefits Schedule in 2002, formalising access to videoconferencing and telephone consultations.^25^ Early evaluations consistently demonstrated benefits, including reduced patient inconvenience and associated costs.^26^ With a long-standing history of virtual healthcare, recent advances mirror global uptake trends and continue to be particularly strategic for overcoming geographical health inequities for rural populations.^27^ Such approaches benefit patients with chronic disease by improving symptom monitoring and access to relevant support despite geographical barriers. Online and remote monitoring devices offer healthcare solutions for consumers to access care without the physical and financial burdens of travelling, providing timely support and allowing for improved and consistent data collection and management.^28^ Consequently, virtual primary healthcare services are increasingly recognised as cost-effective and sustainable models for rural populations across Australia.^29^

The emerging evidence on virtual care models designed for Indigenous populations suggests high user acceptance and satisfaction.^30^ Indigenous-specific and community-controlled digital health interventions are vital for overcoming the distinct barriers faced by rural Indigenous populations, ensuring that care is culturally appropriate, safe and responsive to local needs. However, there remains limited evidence regarding Indigenous engagement and uptake of digital health interventions. Addressing this critical knowledge gap has significant policy implications, as understanding these factors can inform equitable, needs-based implementation strategies aligned with key national priorities in Indigenous health, rural healthcare and national telehealth policy.^31^,^34^

This study is situated within the context of Goondir Health Services, a leading Aboriginal Community Controlled Health Organisation (ACCHO) operating across the regional and rural Darling Downs and South-West Hospital and Health Services (HHS) areas of Queensland, and includes four main clinics in Chinchilla (MMM4 (Modified Monash Model rurality classification^35^), Dalby (MMM4), Oakey (MMM5) and St George (MMM6). Collectively, Goondir provides care to almost 4000 Indigenous clients. According to Goondir’s administrative records in 2024, approximately 63% of active clients were living with at least one chronic health condition. In response to address a consumer need, in 2020, Goondir launched an Indigenous-led VHS programme to enhance care accessibility for rural and remote Indigenous clients with chronic diseases. This VHS model is built on the principles of the *Gayaa Dhuwi (Proud Spirit) Declaration*, prioritising cultural authority, connection to Country, spirituality, kinship networks and community governance. A formative evaluation of consumer perspectives indicated the programme’s strong performance, revealing significant improvements in access, continuity of culturally safe care and enhanced chronic condition self-management.^8^ This primary qualitative evaluation highlighted the positive influence of local Indigenous healthcare providers and led to the critical recommendation that future research should investigate the sustainability and impact of personalised, consumer-centric services to assess the usability and engagement of the VHS programme.

Existing literature has provided limited quantifiable evidence on determinants of sustained VHS engagement among Indigenous populations. Understanding factors associated with utilisation is essential for effective policy and resource allocation. To our knowledge, this is the first study to comprehensively examine VHS device usage patterns and identify associated factors among Indigenous adults with chronic conditions within an ACCHO-led programme.

## The VHS model

Goondir’s VHS framework provides a holistic approach to chronic disease management, with clients provided with digital health devices, supported by community-based health coaches, (Aboriginal Health Workers and support officers) and virtual access to primary care service in the comfort of one’s home. The VHS kit includes four Bluetooth-enabled remote patient monitoring devices: a P3 pulse oximeter (measuring oxygen saturation and pulse rate), BG5 blood glucose monitor (measuring blood glucose levels), blood pressure monitor (measuring systolic and diastolic blood pressure), weighing scale (measuring body weight). All participants received all four monitoring devices; however, actual device use was tailored individually based on clinical assessment by their health coaches and general practitioners. Participants were encouraged to use every device, but to minimise burden, they were guided to regularly use only those devices that were clinically appropriate for their specific chronic conditions. For example, participants without diabetes were not expected to monitor blood glucose routinely. These devices are paired with a mobile application installed on a provided phone or tablet. Eligible participants, identified through multidisciplinary clinical assessment, receive a VHS kit and training on device use. Participants are encouraged to measure and record their vital signs daily, with data transmitted automatically from the mobile app to a secure, digital clinical dashboard accessible only to Goondir VHS staff. Abnormal readings are flagged on the dashboard. Health coaches, who are trained Aboriginal health workers, address the flagged readings, review the data, and facilitate follow-up consultations via secure video or audio calls. Health coaches maintain regular communication with participants through ongoing telephone check-ins and face-to-face home visits, supporting culturally safe, person-centred virtual healthcare delivery.

## Methods

### Study design and setting

This study employed a cross-sectional, prospective survey design with consumers of the Goondir VHS as a key component of the Innovative Digital–IndigeNouS PrImaRy hEalthcare Delivery (ID-INSPIRED) collaborative project funded by the Medical Research Futures Fund (Grant number: APP2023585). Survey data were collected between June 2024 and July 2025. The ID-INSPIRED Governance Committee, comprising Indigenous consumers, community representatives, health leaders and researchers, co-designed the survey instrument to specifically evaluate determinants of the VHS programme, including digital health engagement and remote monitoring device use. The Goondir VHS model, Indigenous governance, leadership structure and operational context have been previously described elsewhere.^8^

### Sampling frame and study participants

The sampling frame included all VHS clients of Goondir Health Services at the time of survey administration, comprising 153 Indigenous adults (as of June 2024) with chronic conditions who were registered users of remote monitoring devices and resided in communities classified within MMM 3–7 (ie, those of <15 000 population through to small and isolated communities categorised as ‘remote’).

This was a complete enumeration study aiming to recruit all 153 VHS clients rather than selecting a calculated sample from a larger population. This approach was chosen as the total VHS client population at Goondir was finite and accessible, making complete enumeration more appropriate than probability sampling. Sample size adequacy was evaluated post hoc using the events-per-variable criterion, with the achieved sample providing 6.7 events per predictor variable, below the ideal 10:1 ratio but acceptable for exploratory analysis.^36 37^ Eligible participants for this study were consenting Indigenous adults (≥18 years) diagnosed with one or more chronic conditions, registered as active VHS users and who provided verbal or written consent to participate. To maximise response rates, we implemented several strategies: Indigenous health coaches with established participant relationships conducted recruitment; multiple contact attempts via telephone and clinic visits were made; flexible survey modes (telephone, face-to-face or self-completion) were undertaken; and culturally appropriate and informed consent processes providing both written and verbal consent options were available.

Data were not collected on the length of time participants had been enrolled in VHS, as the study focused on current engagement patterns over a 2-week recall period rather than longitudinal usage trends.

### Ethical approval

The study received ethical approval from the University of Queensland Human Research Ethics Committee (2022/HE002522). All participants were informed about the study objectives, procedures, and their rights before providing consent. The Indigenous Governance Committee oversaw all data management processes, maintained adherence to cultural and clinical protocols, and provided final approval before dissemination of findings. The study was conducted in accordance with key Indigenous research ethics and governance frameworks, including the CARE Principles for Indigenous Data Governance^38^ and relevant NHMRC Ethical guidelines for Indigenous Health Research,^39 40^ ensuring that Indigenous communities retained authority, control and benefit over the research data and research outcomes.

### Outcome variable

The outcome variable was VHS device usage in the past 2 weeks, categorised as active versus inactive engagement. Participants were asked: ‘Specifically, regarding Goondir’s Virtual Health Service, in the last two weeks, how many times did you use the monitoring equipment or devices provided to you?’ This assessed usage of any of the four devices (pulse oximeter, blood glucose monitor, blood pressure monitor, weighing scale). Response options included ‘Every day,’ ‘2 or 3 times a week,’ ‘Only once in the past two weeks’ and ‘I haven’t used it in the past two weeks.’ Any reported use classified participants as ‘Active VHS Users’; no use classified them as ‘Inactive VHS Users’. This binary classification was chosen for this exploratory study to first establish any engagement vs complete non-engagement in a population where no prior quantitative data on VHS usage existed. The 2-week recall period was chosen to capture recent, habitual usage patterns while minimising potential recall bias.

### Independent variables

Independent variables included demographic, socioeconomic and health-related behavioural characteristics. Demographic factors comprised age, gender, education level, relationship status and geographic rurality based on the MMM classification. Socioeconomic variables included labour force participation and individual yearly income. Health-related behavioural factors encompassed smoking habits, alcohol consumption and physical activity levels. All key predictors were re-categorised into binary variables to ensure adequate cell sizes and maintain statistical power, consistent with established recommendations for logistic regression in small samples.^36^ Geographic rurality was dichotomised as 0 (MMM4 (medium rural towns)) and 1 (MMM5 (small rural towns), MMM6 (remote communities) and MMM7 (very remote communities)). This grouping was necessary because the original distribution, MMM4=26, MMM5=34, MMM6=13 and MMM7=1, resulted in small cell counts that precluded maintaining distinct rural and remote categories. Additionally, VHS health coach visits were dichotomised from count data (ranging from 1 to 52 visits) into a binary variable (0=no visits, 1=any visit). Detailed variable categorisations are presented in online appendix table A1.

### Statistical analysis

This study used descriptive analysis to characterise the Indigenous participants with chronic diseases who received VHS devices, examining demographic, socioeconomic and health-related characteristics. Bivariate analysis using χ^2^ tests (for categorical variables) and independent samples t-tests (for continuous variables) was used to compare participant characteristics between active and inactive users to identify potential factors associated with VHS engagement. Following the bivariate analysis, we performed a descriptive examination of device usage patterns, including cross-tabulation to determine if participants with certain health conditions exhibited preferential usage of clinically relevant monitoring devices. This was followed by multiple logistic regression analysis to identify independent predictors of active VHS usage. To maintain adequate statistical power and avoid overfitting, we limited the multivariable model to seven predictor variables, consistent with guidelines recommending 10–15 events per predictor for logistic regression.^36 37^ Variables were selected a priori based on theoretical relevance from prior literature on digital health engagement^41^,^44^ rather than using automated stepwise selection methods. Multicollinearity among independent variables was assessed using variance inflation factor (VIF) analysis (results not shown). All VIF values were below 1.2, indicating no concerning multicollinearity. All statistical analyses were conducted using regularly updated Stata V.17.0 (StataCorp LLC, College Station, Texas, USA).

## Results

From 153 eligible active VHS clients at Goondir Health Services (as of June 2024), 75 Indigenous participants with chronic disease provided consent and completed the survey, yielding a response rate of 49.0%. After excluding one participant with incomplete data on the VHS device usage, the final analytic sample comprised 74 participants.

Table 1 presents the demographic, socioeconomic, lifestyle and health characteristics of the study participants overall and stratified by VHS user status. The cohort had a mean (SD) age of 58.3 (14.5) years, was predominantly female (77.0%) and had multiple chronic conditions (91.9%). In this cohort, 64.9% of participants resided in small rural, remote or very remote communities (MMM5–7). The participant cohort faced substantial socioeconomic disadvantage, with 77.0% reporting annual individual income below AUD$40 000, a threshold approximating the national median personal income of AUD$41 860 per year for all Australian adults aged 15 years and over (including those unemployed or retired),^45^ and 73.0% unemployed or not in the labour force. Only 17.6% of participants had completed post-secondary education. Nearly two-thirds (n=47, 63.5%) were active VHS users in the 2 weeks prior to data collection, while 36.5% (n=27) were inactive users. Regarding healthcare utilisation, 41.9% of participants had at least one VHS health coach visit in the past 12 months, and 35.1% reported being hospitalised in the past 12 months.

**Table 1:** Participant characteristics overall and by VHS user status (n=74)

Characteristics	Overall (n=74)	Active users (n=47, 63.51%)	Inactive users (n=27, 36.49%)	P value
	**n (%)**	**n (%)**	**n (%)**	
Age (years)				0.04
Mean (SD)	58.3 (14.5)	56.2 (14.5)	62.1 (13.9)	
Age categories, n (%)				0.04
*18–65 years*	49 (66.2)	35 (71.4)	14 (28.6)	
*66 years and older*	25 (33.8)	12 (48.0)	13 (52.0)	
Gender				0.30
*Male*	17 (23.0)	9 (52.9)	8 (47.1)	
*Female*	57 (77.0)	38 (66.7)	19 (33.3)	
Education				0.26
*Secondary or below*	61 (82.4)	37 (60.7)	24 (39.3)	
*Post-secondary*	13 (17.6)	10 (76.9)	3 (23.1)	
Relationship status				
*Unpartnered*	47 (63.5)	32 (68.1)	15 (31.9)	0.28
*Married/partnered*	27 (36.5)	15 (55.6)	12 (44.4)	
Geographic rurality (MMM)**				
*MMM 4 (medium rural*)	26 (35.1)	22 (84.6)	4 (15.4)	0.01
*MMM 5–7 (small rural/remote/very remote*)	48 (64.9)	25 (52.1)	23 (47.9)	
Participation in labour force, n (%)				0.48
*Unemployed/not in labour force*	54 (73.0)	33 (61.1)	21 (38.9)	
*Employed*	20 (27.0)	14 (70.0)	6 (30.0)	
Individual yearly income, n (%)				0.90
*No or low income (<$40 000*)	57 (77.0)	36 (63.2)	21 (36.8)	
*Moderate income ($40 000–$100 000*)	17 (23.0)	11 (64.7)	6 (35.3)	
Smoking habits, n (%)				0.64
*Former smoker/never smoked*	57 (77.0)	37 (64.9)	20 (35.1)	
*Currently smoking*	27 (23.0)	10 (58.8)	7 (41.2)	
Alcohol drinking, n (%)				0.15
*Former drinker or never drunk*	47 (63.5)	27 (57.4)	20 (42.6)	
*Currently drinking*	27 (36.5)	20 (74.1)	7 (25.9)	
Levels of physical activity, n (%)				0.25
*No physical activity*	19 (25.7)	10 (52.6)	9 (47.4)	
*WHO recommended physical activity*	55 (74.3)	37 (67.3)	18 (32.7)	
Number of chronic conditions				0.10
*Single condition*	6 (8.1)	2 (33.3)	4 (66.7)	
*Multiple conditions*	68 (91.9)	45 (66.2)	23 (33.8)	
Any VHS health coach contact (past 12 months)				0.87
*No*	43 (58.1)	16 (37.2)	27 (62.8)	
*Yes*	31 (41.9)	11 (35.5)	20 (64.5)	
Any hospitalisation (past 12 months)				0.20
*No*	48 (64.9)	33 (68.8)	15 (31.2)	
*Yes*	26 (35.1)	14 (53.9)	12 (46.1)	

* Modified Monash Model (MMM): MMM 4=medium rural towns; MMM 5=small rural towns; MMM 6=remote; MMM 7=very remote. Statistical tests: Independent samples t-test for continuous variables; χ2 test for categorical variables.

VHS, virtual health service.

Bivariate analysis comparing characteristics between active and inactive VHS users identified two variables significantly associated with VHS engagement. Age was significantly associated with VHS usage, with active users being younger than inactive users (mean age 56.2 years vs 62.1 years, p=0.04). Among participants aged 18–65 years, 71.4% were active users compared with 48.0% of those aged ≥66 years (p=0.04). Geographic rurality was significantly associated with active usage (p=0.01), with participants residing in medium rural towns (MMM4) demonstrating substantially higher prevalence of active usage (84.6%) compared with those living in small rural, remote or very remote areas (MMM5–7) (52.1%). Despite the high prevalence of multimorbidity, economic disadvantage and limited formal education, these factors along with gender, employment status, health behaviours, health coach visits and hospitalisation were not significantly associated with VHS usage (all p>0.05).

Table 2 provides a descriptive analysis of VHS device usage patterns stratified by chronic condition type. Although Goondir Health Services provides four VHS devices to all participants regardless of their specific chronic conditions, we explored whether participants might preferentially use devices most clinically relevant to their conditions (eg, blood glucose monitors among those with diabetes, blood pressure monitors among those with hypertension). Device usage patterns were relatively consistent across chronic conditions, with 50–70% of participants with each condition using each of the four device types. Among participants with diabetes (n=33), blood glucose monitor usage was 60.6%, comparable to usage rates among those with other conditions (55.6–62.9%). Similarly, participants with hypertension (n=44) did not demonstrate preferentially higher blood pressure monitor usage (61.4%) compared with their usage of other devices (52.3–59.1%) or compared with blood pressure monitor usage among participants with other conditions (57.6–69.4%). This pattern of non-specific device adoption was consistent across all examined conditions.

**Table 2:** Descriptive analysis of VHS device usage patterns across specific chronic conditions

Chronic condition	Device type	Total n	Not used n (%)	Used n (%)
Hypertension (n=44)				
	Blood pressure monitor	44	17 (38.6)	27 (61.4)
	Blood glucose monitor	44	18 (40.9)	26 (59.1)
	Oximeter	44	21 (47.7)	23 (52.3)
	Weight scale	43*	19 (44.2)	24 (55.8)
Diabetes (n=33)				
	Blood pressure monitor	33	14 (42.4)	19 (57.6)
	Blood glucose monitor	33	13 (39.4)	20 (60.6)
	Oximeter	33	16 (48.5)	17 (51.5)
	Weight scale	32*	14 (43.8)	18 (56.3)
Respiratory diseases (n=36)				
	Blood pressure monitor	36	11 (30.6)	25 (69.4)
	Blood glucose monitor	35*	13 (37.1)	22 (62.9)
	Oximeter	36	13 (36.1)	23 (63.9)
	Weight Scale	35*	13 (37.1)	22 (62.9)
Cardiovascular diseases (n=24)				
	Blood pressure monitor	24	8 (33.3)	16 (66.7)
	Blood glucose monitor	24	9 (37.5)	15 (62.5)
	Oximeter	24	9 (37.5)	15 (62.5)
	Weight scale	23*	9 (39.1)	14 (60.9)
Anxiety/depression (n=37)				
	Blood pressure monitor	37	12 (32.4)	25 (67.6)
	Blood glucose monitor	36*	16 (44.4)	20 (55.6)
	Oximeter	37	13 (35.1)	24 (64.9)
	Weight scale	37	12 (32.4)	25 (67.6)

* (1) Sample size varies due to missing data. (2) ‘Used’ combines 2–3 times/week and daily usage. (3) Participants may have multiple chronic conditions. (4) Conditions with n<20 (liver problems n=7, kidney diseases n=12) excluded due to small sample size.

VHS, virtual health service.

**Figure 1:**
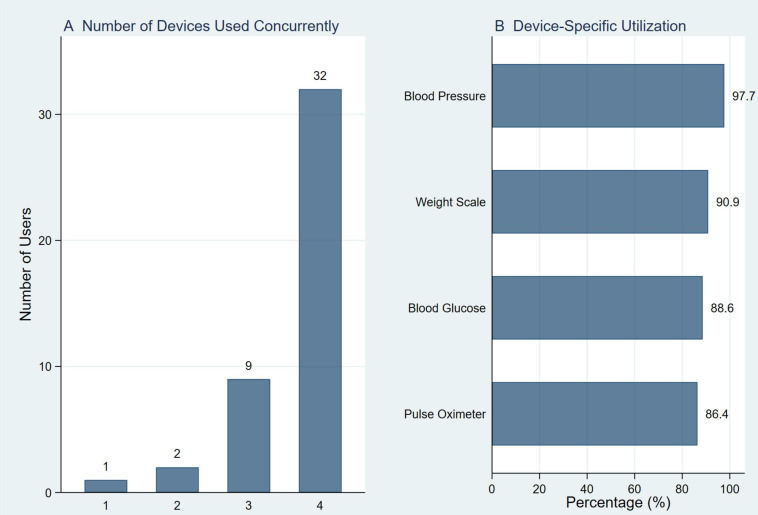
Concurrent and device-specific usage patterns among active virtual health service (VHS) users with complete data (n=44).

Figure 1 presents the device usage patterns among active virtual health service (VHS) users with complete data for all four device types. Panel A shows the extent of concurrent device use during the 2 weeks preceding the survey. Of the 47 active users, 44 (93.6%) had complete data for all device types and were included in this analysis. The majority of users (n=32; 73%) concurrently used all four devices (pulse oximeter, blood glucose monitor, blood pressure monitor, and weight scale). A further 20% (n=9) used three devices, while only a small proportion used two (5%; n=2) or one device (2%; n=1). Panel B shows device-specific utilisation rates among these 44 active users. The blood pressure monitor had the highest uptake (98%), followed by the weight scale (91%), blood glucose monitor (89%), and pulse oximeter (86%). Overall, the figure highlights that nearly all active users (93%) engaged with at least three of the four monitoring devices, demonstrating substantial multi-device engagement.

Table 3 summarises the results of the multivariable logistic regression analysis examining factors associated with VHS device usage. Three factors were significantly associated with VHS engagement after adjusting for covariates. Geographic rurality emerged as the strongest predictor, with participants residing in medium rural towns (MMM4) having significantly higher odds of VHS usage (adjusted OR 4.71, 95% CI 1.23 to 17.94, p=0.02) compared with those in small rural, remote or very remote areas (MMM5–7). Participants aged 18–65 years demonstrated higher odds of VHS usage compared with those aged 66 years and older (adjusted OR 3.59, 95% CI 1.05 to 12.22, p=0.04). Additionally, participants with multiple chronic conditions had significantly higher odds of VHS usage compared with those with a single condition (adjusted OR 10.95, 95% CI 1.25 to 95.87, p=0.03).

**Table 3:** Multivariable analysis of factors associated with VHS device usage (n=74)

Variable	Adjusted OR (95% CI)*	P value
Age		
*18–65 years*	3.59 (1.05 to 12.22)	**0.04**
*66 years and older*	1.00 (reference)	
Gender		
*Male*	1.00 (reference)	
*Female*	1.55 (0.42 to 5.69)	0.50
Education		
*Less than year 12*	1.00 (reference)	0.30
*Year 12 completed*	2.59 (0.43 to 15.57)	
Geographic rurality		
*MMM 4*	4.71 (1.23 to 17.94)	**0.02**
*MMM 5, 6, 7*	1.00 (reference)	
Chronic disease burden		
*Single condition*	1.00 (reference)	
*Multiple condition*	10.95 (1.25 to 95.87)	**0.03**
Any VHS health coach visit (last 12 months), n (%)		
*No*	1.00 (reference)	
*Yes*	1.51 (0.46 to 4.91)	0.49
Any hospitalisation (last 12 months), n (%)		
*No*	3.01 (0.86 to 10.49)	
*Yes*	1.00 (reference)	0.08

* (1) All variables in the bivariate analysis are not included in multivariable model due to limited sample size and model parsimony. (2) OR; CI. (3) Bold p values indicate statistical significance (p<0.05)

VHS, virtual health service.

Sensitivity analyses examined engagement robustness across three intensity thresholds (online appendix table A5). At the ‘any use’ threshold (n=47), age, remoteness and multiple conditions were significant predictors. As engagement intensity increased (≥3 devices: n=45; all 4 devices: n=33), effect sizes diminished and statistical significance weakened, likely due to reduced sample size and statistical power. At the highest threshold, gender became a significant predictor (females OR=4.00, p=0.043), suggesting different factors may influence comprehensive versus minimal device adoption.

We conducted multivariable logistic regression analyses to examine whether specific chronic conditions predicted usage of clinically relevant devices after adjusting for age, gender, education and geographic rurality. Consistent with the descriptive patterns, no specific chronic conditions were significantly associated with their corresponding device usage (all p>0.05) (online appendix table A2). Given the high prevalence of multimorbidity in our sample, we conducted supplementary analyses examining VHS device usage patterns across major chronic condition combinations (online appendix table A3) and their associations with device usage in multivariable models (online appendix table A4). However, these analyses also yielded no significant associations between specific multimorbidity patterns and corresponding device usage, likely reflecting the challenge that comparison groups also predominantly consisted of individuals with chronic conditions, limiting our ability to detect condition-specific effects on device adoption.

## Discussion

This study examined VHS monitoring device usage patterns among rural residing Indigenous adults with chronic conditions receiving care through a primary healthcare ACCHO-controlled entity in rural Queensland, revealing several key findings with important implications for digital health equity. First, active VHS users demonstrated substantial multi-device engagement, with nearly three-quarters using all four monitoring devices concurrently. Among active users, blood pressure monitoring showed the highest utilisation, followed by weight scales, blood glucose monitors and pulse oximeters. Second, chronic disease burden emerged as the strongest predictor of VHS engagement, with participants having multiple chronic conditions demonstrating over 10 times the odds of usage compared with those with a single condition. Third, age was significantly associated with VHS usage, with younger and middle-aged participants (18–65 years) showing nearly four times the odds compared with older adults (66 years and older). Fourth, geographic location remained significantly associated with VHS use, with participants residing in medium rural towns demonstrating four times the odds of usage compared with those in smaller communities and more remote areas.

Chronic disease burden represented the strongest predictor of VHS engagement. This pattern aligns with evidence suggesting that people with several chronic conditions often derive greater perceived value from digital health tools helpful for routine self-management and coordinated care between providers.^46 47^ Consistent with this, telemedicine has been shown to support regular condition monitoring in multimorbid patients through improved access, continuity of care and patient-perceived acceptability.^48^ Broader evidence demonstrates that telemedicine interventions for chronic disease management improve clinical outcomes including glycaemic control, blood pressure reduction and reduced hospitalisation rates.^49^ The higher engagement observed among multimorbid participants suggests the VHS programme has successfully demonstrated this value to those who stand to benefit most. This is noteworthy given that most digital health technologies are designed for single conditions,^50^ yet this programme effectively supported comprehensive monitoring across multiple chronic diseases through an integrated platform. This success underscores the capacity of ACCHOs to implement culturally appropriate digital health innovations that meet the complex, multifaceted needs of clients, directly reflecting the holistic health perspectives central to Indigenous community governance.

Age was significantly associated with VHS engagement, with younger and middle-aged participants (18–65 years) demonstrating nearly four times the odds of usage compared with older adults (≥66 years). This pattern likely reflects greater digital fluency among younger and middle-aged Indigenous adults, who are generally more familiar with smartphones and tablets and thus better able to navigate digital platforms and troubleshoot technical issues independently. Such digital competence likely facilitates seamless integration of VHS monitoring into daily routines, promoting proactive chronic disease self-management. Conversely, older participants often experience lower levels of digital health literacy, making it more difficult to adopt and sustain virtual health engagement.^51 52^ Limited digital skills may therefore act as a structural barrier to accessing virtual care and exacerbate health disparities in later life.^53^ Nonetheless, the lower engagement among older adults presents an opportunity for targeted intervention. Tailored, age-appropriate strategies, such as extended hands-on training, simplified device interfaces, and involvement of family members or carers, could help bridge this gap. Training delivered by trusted Indigenous health coaches, supported by peer mentoring and intergenerational knowledge transfer, may be particularly effective in improving digital health equity across age groups.

This present study also revealed geographic location as a significant predictor of VHS engagement, with participants residing in medium rural towns (MMM4) demonstrating four times the odds of VHS usage compared with those in more remote areas. The higher engagement in less remote settings can be explained by several interrelated factors. A prior ID-INSPIRED study identified that person-centred, culturally appropriate healthcare elements within the VHS model, particularly the vital role of health coaches and the importance of community connections, were central to sustain consumer engagement.^8^ The concentration of VHS staff and health coaches in medium rural towns through this model enables more consistent face-to-face support, follow-up and direct access to clinicians, which facilitates sustained at-home VHS engagement. These settings offer optimal conditions for digital health implementation, including better connectivity infrastructure and more readily accessible technical and clinical support services. Conversely, the lower engagement in the most geographically isolated communities reflects persistent structural and contextual barriers, including workforce challenges. Despite the need, the availability of sufficient locally embedded workforce as part of the VHS model in more remote areas continues to impact healthcare access and service delivery. Many remote Indigenous communities also continue to face systemic deficiencies in telecommunications infrastructure, where service quality, congestion and affordability issues undermine reliable connectivity despite recent investments exceeding $155 million.^54^ A large proportion of Indigenous Australians in these areas rely primarily on mobile internet access, which can further constrain connectivity, particularly when devices are shared among multiple household members.^55^ Prior research has linked limited broadband coverage to lower uptake of digital health tools, and unreliable connections may further erode user confidence, contributing to disengagement from virtual health initiatives.^54 56^ These findings highlight the need for enhanced, targeted infrastructure investment and expanded health coach capacity in remote communities to ensure equitable access to VHS benefits across all geographic contexts.

An important consideration is the clinical appropriateness of device use relative to individual chronic condition profiles. While the Goondir VHS model provides all participants with four monitoring devices, device selection is individually tailored based on clinical assessment, with health coaches guiding participants to use only clinically appropriate devices (eg, participants without diabetes would not monitor blood glucose). However, our study did not assess whether device use aligned with clinical indications. Non-indicated monitoring may pose risks including false positive alerts, increased clinical workload, patient anxiety and discomfort from unnecessary testing.

The clinical utility and monitoring purpose vary substantially by device and condition. Blood pressure and blood glucose monitoring in stable chronic disease primarily serve ongoing disease management rather than acute deterioration detection, as symptomatic acute changes would typically prompt direct healthcare contact. Weight monitoring may provide clinical value for detecting fluid retention in heart failure, liver disease or renal failure, but has limited utility for other conditions. Pulse oximetry is most valuable for respiratory conditions where early desaturation detection may prevent acute decompensation, but routine monitoring without respiratory disease lacks clear clinical benefit.

Nevertheless, the extremely high multimorbidity burden (92% had multiple conditions) means most participants have clinical indications for multiple devices, and logistical challenges in geographically dispersed populations may make comprehensive device provision more practical than multiple condition-specific deliveries. Future research should examine whether device use aligns with clinical indications, whether engagement improves clinical outcomes and the comparative effectiveness of universal versus targeted device provision.

### Strengths and limitations

This study has several notable strengths. To our knowledge, this is the first study to comprehensively examine VHS engagement through a community-based primary healthcare service among rural and remote Indigenous Australians, reporting adoption rates, usage patterns, device preferences and factors associated with engagement. This investigation was conducted within routine clinical practice at an established ACCHO, providing real-world evidence of an Indigenous-led digital health model that enhances external validity and applicability to healthcare decision-making. The study’s focus on objective device usage data rather than self-reported satisfaction measures strengthens the validity of findings and provides reliable indicators of sustained technology adoption. The research partnership with an ACCHO ensured the research was culturally appropriate and guided by Indigenous governance principles, addressing the critical need for culturally responsive digital health evidence. Methodologically, the comprehensive assessment of engagement across four different device types, combined with the inclusion of participants from diverse rural settings, enabled examination of both multi-device usage patterns and geographical disparities in digital health access. The focus on participants with chronic conditions and multimorbidity ensures clinical relevance, as findings directly address the population most likely to benefit from virtual health monitoring.

This study has several limitations. The cross-sectional design precludes causal inference, although planned longitudinal follow-up will address this. Although close to a 50% response rate, the modest sample size (n=74) results in wide CIs for some estimates limits statistical power for certain subgroup comparisons, particularly those with small cell sizes (eg, only six participants with single chronic condition). The 49% response rate may introduce selection bias if more engaged VHS users were more likely to participate, potentially overestimating population-level engagement rates and underestimating barriers to use. We were unable to compare responders with non-responders as data were only available from participants who provided consent and completed the survey, limiting our ability to assess the direction and magnitude of potential non-response bias. Findings may not generalise beyond similar ACCHO-led VHS programmes in regional and remote Queensland contexts. Self-reported device usage is subject to recall and social desirability bias, although the 2-week recall period aimed to minimise this. The binary outcome classification (active/inactive) does not capture variations in engagement intensity or clinical appropriateness of device use; participants with single minimal use (eg, one blood glucose reading in 2 weeks) were classified identically to those with daily comprehensive monitoring across all devices. Future research should develop and validate measures of clinically meaningful engagement appropriate for different chronic conditions. We did not assess whether device use aligned with clinical indications for individual participants’ chronic conditions, limiting our ability to evaluate the clinical appropriateness of monitoring patterns observed. Finally, absence of clinical outcome data limits assessment of VHS clinical effectiveness.

### Implications and future research directions

This study provides preliminary evidence on VHS engagement patterns among rural Indigenous adults with chronic conditions within an established, Indigenous-led ACCHO. However, as a cross-sectional engagement study, these findings cannot determine whether VHS improves health outcomes or justifies implementation recommendations.

Several research priorities emerge from these findings. The substantially higher engagement among participants with multiple chronic conditions validates the programme’s integrated approach, suggesting VHS programmes may benefit from prioritising multimorbid individuals with comprehensive monitoring needs. However, engagement findings alone cannot justify programme expansion. Longitudinal outcome evaluation is essential to determine whether VHS monitoring translates to improved disease control, reduced acute care utilisation, and enhanced quality of life and social-emotional well-being outcomes. Economic evaluation weighing implementation costs, including client time investment and ACCHO resource requirements, against potential health benefits is critical before recommending broader rollout.

Understanding barriers to engagement among underrepresented groups warrants investigation. The substantially lower engagement among older adults and those in more remote communities suggests potential barriers requiring further research. Whether addressing these barriers improves health outcomes remains an empirical question. The role of Indigenous health coaches in facilitating engagement requires systematic evaluation. Understanding how culturally grounded support influences outcomes represents a priority research area. This study was conducted within an established ACCHO with strong community trust and governance structures. Generalisability to other settings, particularly mainstream services, cannot be assumed without research examining implementation factors and outcomes across diverse contexts.

These findings demonstrate feasibility and substantial engagement within a culturally appropriate model, representing an important first step in building an evidence base for VHS in Indigenous primary care. However, outcomes research is required to determine whether engagement translates to meaningful health improvements at acceptable costs before broader implementation can be recommended.

## Conclusion

This study provides the first comprehensive examination of VHS engagement within Indigenous-led primary care in dispersed rural communities, establishing that digital health monitoring is feasible and can achieve meaningful uptake when delivered through trusted ACCHO settings with culturally appropriate support. By identifying multimorbidity, younger age and residence in less remote areas as critical engagement determinants, these findings offer important insights for designing equitable digital health interventions in Indigenous communities. However, feasibility and engagement do not establish effectiveness. Without longitudinal clinical outcome data, questions about whether VHS monitoring improves disease management, reduces hospitalisations, or enhances well-being remain unanswered. Before broader implementation, outcomes-focused research and economic evaluation are essential to determine whether the observed engagement translates to health improvements justifying the investment required from both clients and health services. This work establishes a foundation for such investigations while identifying priority areas for future research.

## Supplementary material

10.1136/bmjopen-2025-115212 online supplemental file 1

## Data Availability

Data are available upon reasonable request.
