# Supplementary material for: Patterns and determinants of primary care virtual health service use among rural Aboriginal and Torres Strait Islander adults with chronic diseases: a cross-sectional study
Source: BMJ Open. 2026 May 28;16(5):e115212. doi: 10.1136/bmjopen-2025-115212 (PMC13223628; doi:10.1136/bmjopen-2025-115212)
Supplement: online supplemental file 1 [file bmjopen-16-5-s001.docx]

**Appendix**

**Table A1: Description of key variables and their measurements**

| **Variable** | **Code** | **Category** | **Description** |
| --- | --- | --- | --- |
| ***Outcome variable*** |  |  |  |
| VHS device usage in the past 2 weeks | 0 | Inactive user | Participants did not use any of the four VHS devices in the last 2 weeks |
|  | 1 | Active user | Participants used at least any of the four VHS devices in the last 2 weeks |
| ***Independent variable*** |  |  |  |
| Age | 0 | ≤65 years | Participants aged 18-65 years |
|  | 1 | ≥ 66 years | Participants aged 66 years and older |
| Gender | 0 | Male | Male participants |
|  | 1 | Female | Female participants |
| Education level | 0 | Secondary or below | Never attended school, Year 8 or below, Year 9-11, Year 12 or equivalent |
|  | 1 | Post-secondary | Diploma/vocational/TAFE, University degree |
| Relationship status | 0 | Unpartnered | Never married, Single, Separated, Divorced, Widowed |
|  | 1 | Partnered | Married participants |
| Geographic rurality | 0 | MMM 4 | MMM4 (Medium rural towns) |
|  | 1 | MMM 5,6,7 | MMM5 (Small rural towns), MMM6 (Remote communities), MMM7 (Very remote communities) |
| Participation in labour force | 0 | Employed | Participants who are currently employed |
|  | 1 | Unemployed or not in the labour force | Participants who are unemployed or not participating in the labour force |
| Individual yearly Income | 0 | No or Low income (<$40k) | Participants with no income or income less than $40,000 |
|  | 1 | Moderate income ($40k-$100k) | Participants with income between $40,000 and $100,000 |
| Smoking habits | 0 | Former smoker/Never smoked | Participants who have never smoked or are former smokers |
|  | 1 | Currently smoking | Participants who are currently smoking |
| Alcohol drinking | 0 | Former drinker or never drunk | Not currently drinking (Abstinent) |
|  | 1 | Currently drinking | Currently drinks alcohol |
| Levels of physical activity | 0 | No physical activity | Do not do any physical activity |
|  | 1 | Recommended Physical activity* | At least 150 minutes of moderate-intensity physical activity per week |
| Number of chronic diseases | 0 | Single condition | Participants with one self-reported chronic disease condition |
|  | 1 | Multiple condition | Participants with multiple self-reported chronic disease conditions |
| Any VHS health coach visit (last 12 months) | 0 | No | No VHS health coach visits in the past 12 months |
|  | 1 | Yes | At least one VHS health coach visit in the past 12 months |
| Any hospitalisation (last 12 months) | 0 | No | No hospitalization in the past 12 months |
|  | 1 | Yes | At least one hospitalization in the past 12 months |

Notes: 1. *Physical activity levels were categorized according to the World Health Organization (WHO) recommendations for adults, which suggest engaging in at least 150 minutes of moderate-intensity physical activity per week.

**Table A2: Multivariable logistic regression models examining chronic condition associations with VHS device engagement**

| **Model 1 Blood Pressure Monitor**  **(Not regular user vs regular user)** | | |  | **Model 2 Blood Glucose Monitor**  **(Not regular user vs regular user)** | |
| --- | --- | --- | --- | --- | --- |
|  | AOR  (95% CI); p-value | |  |  | AOR  (95% CI); p-value |
| **Hypertension** |  | |  | **Diabetes** |  |
| No | 1.00 (reference) | |  | No | 1.00 (reference) |
| yes | 1.75 (0.51-5.63); p = 0.36 | |  | yes | 1.62 (0.55-4.82); p = 0.38 |
|  | | | | | |
| **Model 3 Oximeter**  **(Not regular user vs regular user)** | | |  | **Model 4 Weight Scale**  **(Not regular user vs regular user)** | |
|  | | AOR  (95% CI); p-value |  |  | AOR  (95% CI); p-value |
| **Respiratory Problems** | |  |  | **Heart Problems** |  |
| No | | 1.00 (reference) |  | No | 1.00 (reference) |
| yes | | 1.57 (0.56-4.41); p = 0.39 |  | yes | 1.13 (0.37-3.48);p = 0.83 |

Notes: 1. All models adjusted for age (≤65 vs 66+ years), gender (male vs female), education (secondary or below vs post-secondary), and geographic rurality (medium rural town vs small rural/remote/very remote communities), any VHS coach visit in last 12 month (No vs Yes), and any hospitalisation in last 12 month (No vs yes); 2. OR = Odds Ratio; CI = Confidence Interval

**Table A3:** **Descriptive Analysis of VHS Device Usage Patterns across Major Chronic Condition Combinations (hypertension, diabetes, heart problems, or respiratory conditions)**

| **Device Type** | **Single Major Condition^a^** | **Hypertension + Diabetes** | **Heart + Hypertension** | **Respiratory + Hypertension** | **Other Combinations^d^** | **p-value^f^** |
| --- | --- | --- | --- | --- | --- | --- |
|  | **(n=17)** | **(n=24)** | **(n=8)** | **(n=7)** | **(n=18)** |  |
| **Blood Pressure Monitor**  **(Regular user)** | 10 (58.80%) | 14 (58.30%) | 7 (87.50%) | 5 (71.40%) | 11 (61.10%) | 0.61 |
| **Blood Glucose Monitor**  **(Regular user)** | 9 (52.90%) | 14 (58.30%) | 7 (87.50%) | 4 (57.10%) | 8 (47.10%)^e^ | 0.42 |
| **Oximeter**  **(Regular user)** | 8 (47.10%) | 13 (54.20%) | 6 (75.00%) | 4 (57.10%) | 11 (61.10%) | 0.74 |
| **Weight Scale**  **(Regular user)** | 9 (56.30%)^b^ | 13 (56.50%)^c^ | 6 (75.00%) | 4 (57.10%) | 11 (61.10%) | 0.91 |

Notes: 1. regular use defined as ≥2 times per week usage frequency; a. Single major condition includes participants with only one of the four major chronic conditions (hypertension, diabetes, heart problems, or respiratory conditions) as their primary condition, though they may have additional minor chronic conditions; b. Weight scale n=16 for single major condition due to missing data; c. Weight scale n=23 for hypertension + diabetes due to missing data; d. Complex combinations include participants with ≥3 major chronic conditions or other specific combinations not fitting the above categories; e. Blood glucose monitor n=17 for complex combinations due to missing data; f. Chi-square test comparing device usage across major condition combinations

**Table A4:** **Multivariable Logistic Regression of Multimorbidity Patterns and VHS Device Usage**

|  | **BP Monitor**  **(Regular user vs Not regular user)** | **p-value** | **Glucose Monitor**  **(Regular user vs Not regular user)** | **p-value** | **Oximeter**  **(Regular user vs Not regular user)** | **p-value** | **Weight Scale**  **(Regular user vs Not regular user)** | **p-value** |
| --- | --- | --- | --- | --- | --- | --- | --- | --- |
|  | **Model 1** |  | **Model 2** |  | **Model 3** |  | **Model 4** |  |
| **Multimorbidity Pattern** | **AOR (95% CI)** |  | **AOR (95% CI)** |  | **AOR (95% CI)** |  | **AOR (95% CI)** |  |
| *Single condition* | 1.00 (ref) |  | 1.00 (ref) |  | 1.00 (ref) |  | 1.00 (ref) |  |
| *Hypertension + Diabetes* | 1.63 (0.39-7.88) | 0.54 | 1.91 (0.39-9.26) | 0.42 | 2.36 (0.51-10.84) | 0.27 | 1.80 (0.37-8.73) | 0.46 |
| *Heart + Hypertension* | 10.40 (0.76-142.70) | 0.08 | 12.22 (0.93-160.89) | 0.06 | 5.94 (0.68-51.86) | 0.11 | 4.22 (0.49-35.89) | 0.19 |
| *Respiratory + Hypertension* | 3.17 (0.37- 27.20) | 0.29 | 2.05 (0.28-14.90) | 0.48 | 2.28 (0.32-16.38) | 0.42 | 1.51 (0.22-10.49) | 0.68 |
| *Complex multimorbidity* | 0 .98 (0.20-4.73) | 0.98 | 0.56 (0.12-2.68) | 0.47 | 1.75 (0.39-7.84) | 0.46 | 1.31 (0.28-6.02) | 0.73 |
| **Geographic rurality** |  |  |  |  |  |  |  |  |
| *Medium rural town* | 4.40 (1.20-16.15) | **0.02** | 5.00 (1.41-17.65) | **0.01** | 2.23 (0.71-7.07) | 0.17 | 3.06 (0.95-9.83) | 0.06 |
| *Small rural towns/ Remote/very remote communities* | 1.00 (reference) |  | 1.00 (reference) |  | 1.00 (reference) |  | 1.00 (reference) |  |

Notes: 1. All models adjusted for age (≤65 vs 66+ years), gender (male vs female), education (secondary or below vs post-secondary), and geographic rurality (medium rural town vs small rural/remote/very remote communities), any VHS coach visit in last 12 month (No vs Yes), and any hospitalisation in last 12 month (No vs yes); 2. Bold p-values indicate statistical significance (p<0.05); OR = Odds Ratio; CI = Confidence Interval

**Appendix Table A5: Sensitivity Analysis - Predictors of VHS Engagement at Different Usage Thresholds (n=74)**

| **Variable** | **Any Use** |  | **≥3 Devices** |  | **All 4 Devices** |  |
| --- | --- | --- | --- | --- | --- | --- |
|  | **Adjusted OR (95% CI)** | **p-value** | **Adjusted OR (95% CI)** | **p-value** | **Adjusted OR (95% CI)** | **p-value** |
| Sample size (active users) | n=47 (63.5%) |  | n=45 (60.8%) |  | n=33 (44.6%) |  |
| **Age** |  |  |  |  |  |  |
| *18-65 years* | **3.59 (1.05-12.22)** | **0.04** | 2.82 (0.87-9.12) | 0.08 | 2.77 (0.86-8.92) | 0.09 |
| *66 years and older* | 1.00 (reference) |  | 1.00 (reference) |  | 1.00 (reference) |  |
| **Gender** |  |  |  |  |  |  |
| *Male* | 1.00 (reference) |  | 1.00 (reference) |  | 1.00 (reference) |  |
| *Female* | 1.55 (0.42-5.69) | 0.51 | 2.93 (0.84-10.20) | 0.09 | **4.00 (1.04-15.31)** | **0.04** |
| **Education** |  |  |  |  |  |  |
| *Secondary or below* | 1.00 (reference) |  | 1.00 (reference) |  | 1.00 (reference) |  |
| *Post-secondary* | 2.60 (0.43-15.57) | 0.30 | 1.45 (0.31-6.90) | 0.64 | 1.33 (0.32-5.52) | 0.69 |
| **Geographic rurality** |  |  |  |  |  |  |
| *MMM 4* | **4.71 (1.23-17.94)** | **0.02** | 2.61 (0.78-8.68) | 0.12 | 1.29 (0.43-3.88) | 0.65 |
| *MMM 5,6,7* | 1.00 (reference) |  | 1.00 (reference) |  | 1.00 (reference) |  |
| **Chronic disease burden** |  |  |  |  |  |  |
| *Single condition* | 1.00 (reference) |  | 1.00 (reference) |  | 1.00 (reference) |  |
| *Multiple conditions* | **10.95 (1.25-95.87)** | **0.03** | 6.72 (0.82-55.14) | 0.08 | 2.97 (0.35-25.16) | 0.31 |
| **Any VHS health coach visit (last 12 months)** |  |  |  |  |  |  |
| *No* | 1.00 (reference) |  | 1.00 (reference) |  | 1.00 (reference) |  |
| *Yes* | 1.51 (0.46-4.92) | 0.50 | 1.35 (0.43-4.24) | 0.60 | 0.52 (0.17-1.55) | 0.24 |
| **Any hospitalization (last 12 months)** |  |  |  |  |  |  |
| *No* | 3.00 (0.86-10.49) | 0.09 | 3.09 (0.93-10.26) | 0.07 | 2.78 (0.86-9.01) | 0.09 |
| *Yes* | 1.00 (reference) |  | 1.00 (reference) |  | 1.00 (reference) |  |
|  |  |  |  |  |  |  |

Notes: 1. Any use = used at least 1 device in past 2 weeks; ≥3 devices = used 3 or more devices regularly (≥2-3 times/week); All 4 devices = used all 4 monitoring devices regularly. 2. The ≥2 devices threshold yielded nearly identical sample size (n=47) to 'any use', differing by only 2 participants with inconsistent survey responses, and is therefore not presented separately. 3. All models adjusted for all variables shown in the table. 4. Bold p-values indicate statistical significance (p<0.05). 5. OR = Odds Ratio; CI = Confidence Interval; MMM = Modified Monash Model. 6. As engagement threshold increases, effect sizes diminish and statistical significance weakens, likely due to reduced sample size and statistical power.
