# Supplementary material for: Patterns and determinants of primary care virtual health service use among rural Aboriginal and Torres Strait Islander adults with chronic diseases: a cross-sectional study
Source: BMJ Open. 2026 May 28;16(5):e115212. doi: 10.1136/bmjopen-2025-115212 (PMC13223628; doi:10.1136/bmjopen-2025-115212)
Supplement: Supplementary file 2 [file bmjopen-2025-115212.reviewer_comments.pdf]

PEER REVIEW HISTORY

BMJ Open publishes all reviews undertaken for accepted manuscripts. Reviewers are asked to complete a checklist review form and are provided with free text boxes to elaborate on their assessment. These free text comments are reproduced below.

ARTICLE DETAILS

Title (Provisional)

Patterns and Determinants of Primary Care Virtual Health Service Use Among Rural Aboriginal and Torres Strait Islander Adults with Chronic Diseases: A Cross-Sectional Study

Authors

Haque, Rezwanul; Luebbe, Annika; McGrail, Matthew R.; Nasir, Bushra F; Alam, Khorshed; Wallis, Katharine; Leedie, Floyd; Kondalsamy-Chennakesavan, Srinivas

---

VERSION 1 - REVIEW

**Reviewer**	**1**
**Name**	**Murdaca, Giuseppe**
**Affiliation**	**University of Genoa**
**Date**	**01-Mar-2026**
**COI**	**None**

The paper is interesting and and well written. The authors investigated virtual health service (VHS) device usage patterns and identify factors associated with VHS engagement among Indigenous Australian adults with chronic conditions. The participants completed surveys assessing their use of four Bluetooth-enabled

monitoring devices (pulse oximeter, blood glucose monitor, blood pressure monitor, weight scale) over a two-week recall period. The study confirmed that sustaining and expanding these benefits requires investment in digital connectivity and Indigenous workforce development. The paper may be published in current form.

**Reviewer**	**2**
**Name**	**Zanker, Jesse**
**Affiliation**	**University of Melbourne, Department of Medicine - Royal Melbourne Hospital**
**Date**	**30-Mar-2026**

COI **None**

Patterns and Determinants of Primary Care Virtual Health Service Use Among Rural Aboriginal and Torres Strait Islander Adults with Chronic Diseases

Review

General comment

This is an important study of engagement in remote monitoring among Aboriginal and Torres Strait Islander adults. The paper is well written and executed. The study is novel and an important step in understanding the role of digital technologies in health care in a priority population. The conclusions drawn, however, extend beyond what the study is designed to show (being cross-sectional in nature and based on engagement, not outcomes). The paper requires revision of the interpretation and implications of the findings. However, I think with revision the paper would meet expected standards for publication and holds important hypothesis-generating findings for future study.

Abstract

- Generally clear and succinct

- The conclusion brings new information – no mention of culturally grounded health coach role in the study prior to the conclusion. Did inactive users not have access to the health coach?

- The conclusion also assumes there is health benefit from VHS. The study hasn't assessed whether there is health benefit, it has assessed whether there is use. Monitoring something doesn't automatically equate to health benefits. There are many other factors; what if there are no services available to respond? Or can't get into the GP/ALHO at the ACCHO? Afterhours? Will that cause more stress, if the result is true but nonsignificant, or an aberration? Consider restraining the conclusions in the abstract.

Strengths and limitations

- Consider splitting the second dot point as the two statements seems distinct, linked only by semi colon.

Introduction

- Sets up the study well. Clearly written.

- Minor point. The term VHS when referring to digital health is a bit confusing to read, as an older reader's mind reverts to thinking this acronym is referring to the analogue, Video Home System. I suppose this is unavoidable.

- Only the positive findings of telehealth are discussed; there are many limitations and some clinical risks, including device/tech literacy, sensory impairment in older peoples, which should also be mentioned in the introduction. VHS is not a panacea as indicated here.

- Typo page 5 line 37.

- Good explanation of what occurs when results are flagged; this understanding provides more context to the conclusions drawn in the abstract.

Methods

- The Outcome variable of “active user,” that is any use of any device in the last 2 weeks, may set the threshold too low to determine clinically useful use. A single BSL, for example, once in 2 weeks for a person with diabetes may be very problematic. Compared with a generally well person with osteoarthritis alone, who is checking all four items every day.

- Could a sensitivity analysis examine levels of usage in more detail? To determine if the overall findings remain robust? Not critical for publication but helpful for interpretation.

- Statistical analysis appropriate and well justified.

Results

- Page 11 line 6 – provide some context regarding the 40K annual income (where this sits compared to national average), also include AUD for international readers.

- Table 1 and 2 could probably be combined, with columns total / active users / inactive users / p-value. Having these tables spilt doesn’t add much.

- As the figure / venn diagram is not scaled it is a little difficult to interpret. Consider scaling it to the proportions represented by the fields.

Discussion

- Page 12 line 45 regarding effectiveness of telemedicine: how is the effectiveness measured? User satisfaction or outcomes? Please clarify.

- The observation of non-specific adoption of devices regardless of personalised need is a risk of this approach. Routine 02 monitoring is not recommended in persons without respiratory symptoms or problems and could result in significant unintended health service burden if there are false positives (common due to factors such as nail polish). Further, there is an ethical dilemma in monitoring people for conditions they don’t have, adding to my reservations about monitoring everyone with all four machines in the same way, regardless of degree of multimorbidity. It is also more resource intensive to do so, where these resources could be better targeted and spread across more people. For example, if someone had type 2 diabetes and hypertension, the BP and BSL monitors would be appropriate, but perhaps not scales and oximeter. If someone had severe COPD and was cachectic and a CO2 retainer, then perhaps the oximeter and weight scales would be appropriate, but not BP and BSL monitor. There is also pain / discomfort associated with monitoring BSLs / BP when not required, so this should be addressed in the discussion. We don’t wish to cause discomfort to people unless it is advisable on the balance of risk/benefit for their health.

- Those with higher use were in medium towns; the opposite of what might be hoped (more remote have less access to health care). This is discussed well.

- It is not quite clear whether the monitoring is for acute or subacute purposes. For example, BSL, BP and 02 may be useful to detect acute deterioration, however clients are likely to be symptomatic (excepting high BP until extreme) which would trigger assessment anyway. Weight scales may only be beneficial in heart/liver/kidney failure population to detect fluid retention acute/subacutely, but may have limited benefit in other populations. A nuanced discussion of how targeted monitoring might be more effective, rather than a blanketed approach, would assist the manuscript.

- Page 15 line 19. While I would be guided by the Indigenous co-authors and governance group, I'm not sure that multi-device platforms is akin to "Indigenous holistic health perspectives," which may be less guided by specific health conditions alone, but rather in concert with connection to inner spirit, community and Country (see Good Spirit Good Life literature). Consider phrasing this differently.

- I don't think the described implications for policy and practice are justified based on the findings of this cross-sectional study. This is a usage-based study, not outcome. To roll this out further, at significant cost to clients (through time spent) and ACCHOs, there would need to be a longitudinal outcomes-based assessment. Does this change / improve health and social/emotional wellbeing outcomes? This question cannot be answered by the cross-sectional design in this study.

Conclusion

- The conclusion repeats the findings in the first para of the discussion – it could be made more succinct.

- I don't believe the conclusions made are supported by this study. Asserting expanding workforce capacity and roll out on the basis of an engagement study does not yet demonstrate benefit; it's promising, but further longitudinal, outcomes based study is required. Otherwise there is a risk that large human and financial resources are used on something that has high engagement, but doesn't improve outcomes. Therefore, I think the conclusions need to reflect the study findings and point towards further study including cost/benefit analysis before considering wider rollout.

VERSION 1 - AUTHOR RESPONSE

**Reviewer 1**			
**Reviewer's comments**		**Author's response**	**References**
1	The paper is interesting and well written. The authors investigated virtual health service (VHS) device usage	We thank Reviewer 1 for their supportive feedback. No action required.	N/A

	patterns and identify factors associated with VHS engagement among Indigenous Australian adults with chronic conditions. The participants completed surveys assessing their use of four Bluetooth-enabled monitoring devices (pulse oximeter, blood glucose monitor, blood pressure monitor, weight scale) over a two-week recall period. The study confirmed that sustaining and expanding these benefits requires investment in digital connectivity and Indigenous workforce development. The paper may be published in current form.		
**Reviewer-2**			
**Reviewer's comments**		**Author's response**	**References**
1.	This is an important study of engagement in remote monitoring among Aboriginal and Torres Strait Islander adults. The paper is well written and executed. The study is novel and an important step in understanding the role of digital technologies in health care in a priority population. The conclusions drawn, however, extend beyond what the study is designed to show (being cross-sectional in nature and based on engagement, not outcomes). The paper requires revision of the interpretation and	We sincerely thank Reviewer 2 for their thoughtful, detailed, and constructive feedback. This feedback was extremely beneficial, and we have made necessary revisions to the manuscript.	N/A

	implications of the findings. However, I think with revision the paper would meet expected standards for publication and holds important hypothesis-generating findings for future study.		
	**Abstract**		
2.	Generally clear and succinct	Thank you.	
3.	The conclusion brings new information – no mention of culturally grounded health coach role in the study prior to the conclusion. Did inactive users not have access to the health coach?	Thank you for your query. All participants (both active and inactive users) had access to Indigenous health coaches, as described in the Introduction section ("Health coaches, who are trained Aboriginal Health Workers, address the flagged readings, review the data, and facilitate follow-up consultations..."). We have added clarification in the Abstract Methods section to ensure health coach support is introduced before being mentioned in the Abstract Conclusion. In addition, we added in further details within the Virtual Health Services Model section to clarify the role and scope of the health coaches. This is a universal component of the Goondir VHS model provided to all registered VHS clients. The health coach support referenced in the conclusion reflects this Indigenous-led service delivery model rather than a variable distinguishing active from inactive users.	Please refer to methods sub-section of the Abstract<br>Page 2, lines: 29<br><br>Please also refer to 'The Virtual Health Service Model' section after the introduction<br><br>Page 7, lines: 35-42
4.	The conclusion also assumes there is health benefit from VHS. The study hasn't assessed whether there is health benefit, it has assessed whether there is use. Monitoring something doesn't automatically equate to health benefits. There are many other factors; what if there are no services available to respond? Or can't get into the GP/ALHO at the ACCHO? Afterhours? Will that cause more stress, if the result is true	We agree and thank the reviewer for this observation. We have revised the abstract conclusion to focus on what we measured (engagement) rather than implying health benefits.<br><br>**After revision, the Abstract conclusion is now written as:**<br><br>Indigenous-led VHS achieve substantial engagement through culturally grounded health coach support. However, addressing digital connectivity in remote areas, age-appropriate support for older adults, and Indigenous workforce development is essential to ensure equitable access and sustained engagement.	Please refer to conclusion sub-section of the Abstract<br>Page 2, lines: 49-54

	but nonsignificant, or an aberration? Consider restraining the conclusions in the abstract.		
	**Strengths and limitations**		
5.	Consider splitting the second dot point as the two statements seems distinct, linked only by semi colon.	Thank you for guiding us. We have split the second bullet point into two separate points.<br><br>**After revision, it is now written as:**<br>"► Real-world evidence from established practice, rather than experimental pilot conditions, enhances external validity and applicability to similar Aboriginal Community Controlled Health Organisation settings.<br>► Comprehensive assessment across four device types enabled examination of multi-device usage patterns and geographic disparities in digital health access."	Please refer to 'Strength and limitations of this Study section'<br><br>Page 3
	**Introduction**		
6.	Sets up the study well. Clearly written.	Thank you.	N/A
7.	Minor point. The term VHS when referring to digital health is a bit confusing to read, as an older reader's mind reverts to thinking this acronym is referring to the analogue, Video Home System. I suppose this is unavoidable.	We appreciate this observation and acknowledge the potential for confusion with the Video Home System acronym. To address this ambiguity, we have expanded the definition of Virtual Health Services (VHS) when first introduced in the Introduction to clarify what it encompasses within the digital health context: remote digital health monitoring using connected devices to transmit patient-generated health data to healthcare providers for ongoing chronic disease management.	Please refer to the third paragraph in the Introduction section.<br><br>Page: 4; lines: 37-38
8.	Only the positive findings of telehealth are discussed; there are many limitations and some clinical risks, including device/tech literacy, sensory impairment in older peoples, which should also be mentioned in the introduction. VHS is not a panacea as indicated here.	Thank you for your suggestion. We have now added a standalone paragraph to the Introduction, providing a more balanced background of its potential positive and negative aspects.<br><br>**After revision, it is now written as:**<br>However, the evidence base also highlights that VHS is not a universal solution, and a number of well-documented barriers and clinical risks temper its potential. Low digital literacy represents a significant challenge, particularly among older adults, individuals with lower educational levels, and rural and Indigenous populations, where limited familiarity with technology has been shown to substantially reduce successful engagement [3,4]. For older	Please refer to the Introduction section<br><br>Page 5, lines: 3-34

		populations specifically, age-related sensory impairments, including hearing and vision loss, present additional barriers to effective virtual consultation, with studies reporting that such limitations compromise communication quality and increase the likelihood of clinically important information being overlooked during remote encounters [5,6]. Structural inequities in digital infrastructure further constrain access, with inadequate broadband, limited device ownership, and poor internet connectivity in rural areas constituting persistent barriers to VHS uptake [7,8]. At the clinical level, virtual care introduces inherent limitations in the capacity to conduct physical examinations, raising concerns about diagnostic accuracy, incomplete assessment, and patient safety, particularly for complex or new presentations [9,10]. Data privacy, cybersecurity, and the absence of standardised clinical governance frameworks have also been identified as risks associated with virtual care at scale [10,11]. Despite these challenges, telehealth infrastructure and policy frameworks have continued to develop in Australia, creating opportunities to examine how these barriers and facilitators shape actual utilisation patterns in community-controlled health settings.	
9.	Typo page 5, line 37	We have corrected this typo.	Please refer to Page 6, line: 17
10.	Good explanation of what occurs when results are flagged; this understanding provides more context to the conclusions drawn in the abstract.	Thank you.	N/A
	**Method**		
11.	The Outcome variable of “active user,” that is any use of any device in the last 2 weeks, may set the threshold too low to determine clinically useful use. A single BSL, for example, once in 2 weeks for a person with diabetes may be very problematic.	This is an excellent methodological point, and we agree that our binary outcome (any vs. no use) does not capture clinically meaningful usage intensity. However, we chose this binary classification as this is an exploratory, first quantitative study in this population where sample size constraints limited more granular analyses. Our goal was to identify any engagement as a first step, particularly given that clinical guidelines for optimal monitoring frequency are unclear for multi-condition populations. However, we fully	Please refer to ‘Outcome variable’ sub-section in the Method section.<br>Page 9, lines: 45-49

	Compared with a generally well person with osteoarthritis alone, who is checking all four items every day.	acknowledge this limitation and have addressed it by adding explicit acknowledgment in the Methods section and expanding discussion in the Limitations section. Our existing supplementary analyses (Appendix Tables A2-A4) provide additional detail on device-specific usage patterns and intensity among active users.<br><br>**After revision in the Methods section, the manuscript is now written as:**<br>This binary classification was chosen for this exploratory study to first establish any engagement versus complete non-engagement in a population where no prior quantitative data on VHS usage existed.<br><br>**After revision in the Limitations section, the manuscript is now written as:**<br>The binary outcome classification (active/inactive) does not capture variations in engagement intensity or clinical appropriateness of device use; participants with single minimal use (e.g., one blood glucose reading in two weeks) were classified identically to those with daily comprehensive monitoring across all devices. Future research should develop and validate measures of clinically meaningful engagement appropriate for different chronic conditions.	Please also refer to 'Strength and limitations sub-section ' in the discussion section.<br>Page 17, lines: 56-60<br>Page 18, lines: 3-7
12.	Could a sensitivity analysis examine levels of usage in more detail? To determine if the overall findings remain robust? Not critical for publication but helpful for interpretation.	Thank you for your valuable feedback. We conducted sensitivity analyses examining VHS engagement across three intensity thresholds: any device use (n=47, 63.5%), use of ≥3 devices (n=45, 60.8%), and use of all 4 devices (n=33, 44.6%).<br>The ≥2 devices threshold yielded nearly identical sample size (n=47) to 'any use', differing by only 2 participants with inconsistent survey responses between summary and device-specific usage questions, and is therefore not presented separately.<br><br>Results (Appendix Table A5) demonstrate that the three factors, younger age, lower geographic remoteness, and multiple chronic conditions, predict engagement at the 'any use' threshold with statistical significance. As the engagement threshold increases, effect sizes diminish and statistical significance weakens, likely due to reduced sample size and statistical power to evaluate observed differences. At the highest	Please refer to Result section.<br>Page 13, lines: 12-23<br><br>Please also see Appendix Table A5 in the supplementary material

		threshold (all 4 devices), gender emerged as a significant predictor (females OR=4.00, p=0.043), suggesting different factors may influence comprehensive versus minimal device adoption. These findings indicate that while the same factors predict initial VHS engagement, sustaining high-intensity multi-device use may be influenced by additional factors, though larger studies are needed to confirm these patterns. It is now mentioned in the result section.<br><br>**After revision in the Result section, it is now written as:**<br>Sensitivity analyses examined engagement robustness across three intensity thresholds (Appendix Table A5). At the 'any use' threshold (n=47), age, remoteness, and multiple conditions were significant predictors. As engagement intensity increased (≥3 devices: n=45; all 4 devices: n=33), effect sizes diminished and statistical significance weakened, likely due to reduced sample size and statistical power. At the highest threshold, gender became a significant predictor (females OR=4.00, p=0.043), suggesting different factors may influence comprehensive versus minimal device adoption.	
13.	Statistical analysis appropriate and well justified.	Thank you.	N/A
	**Results**		
14.	Page 11 line 6 – provide some context regarding the 40K annual income (where this sits compared to national average), also include AUD for international readers.	We appreciate your suggestion. We have added currency specification (AUD) and contextual comparison to the Australian national median personal income to provide international readers with appropriate context.<br><br>**After revision in the Result section, it is now written as:**<br>The participant cohort faced substantial socioeconomic disadvantage, with 77.0% reporting annual individual income below AUD $40,000, a threshold approximating the national median personal income of AUD $41,860 per year for all Australian adults aged 15 years and over (including those unemployed or retired) [12]	Please refer to the result section.<br><br>Page 11, lines: 21-26
15.	Table 1 and 2 could probably be combined, with columns total / active users / inactive users / p-value. Having	We have now combined Table 1 and 2.	Please refer to Page 27

	these tables spilt doesn't add much.		
16.	As the figure / venn diagram is not scaled it is a little difficult to interpret. Consider scaling it to the proportions represented by the fields.	We agree that the unscaled Venn diagram was difficult to interpret. We have replaced it with a clearer two-panel bar chart that presents the same information in a more accessible format.	Please refer to figure 1.<br>Page 31<br><br>For the description of the figure, please refer to Page 15, lines: 10-20
	**Discussion**		
17.	Page 12 line 45 regarding effectiveness of telemedicine: how is the effectiveness measured? User satisfaction or outcomes? Please clarify.	Thank you for this important point. We have revised the text to clarify that reference 50 (Buawangpong et al., 2024) relates to patient-perceived acceptability of telemedicine, not clinical effectiveness. To address the question of measured clinical effectiveness, we have added a new reference 51 (Kraef et al., 2020), a systematic review and meta-analysis demonstrating that telemedicine interventions for chronic disease management improve clinical outcomes including glycaemic control, blood pressure reduction, and reduced hospitalisation rates.<br><br>**After revision in the Discussion section, it is now written as:**<br><br>Consistent with this, telemedicine has been shown to support regular condition monitoring in multimorbid patients through improved access, continuity of care, and patient-perceived acceptability [13]. Broader evidence demonstrates that telemedicine interventions for chronic disease management improve clinical outcomes including glycaemic control, blood pressure reduction, and reduced hospitalisation rates [14]. The higher engagement observed among multimorbid participants suggests the VHS program has successfully demonstrated this value to those who stand to benefit most.	Please refer to the Discussion section.<br><br>Page 14, lines: 35-46
18.	The observation of non-specific adoption of devices regardless of personalised need is a risk of this approach. Routine 02 monitoring is not recommended in persons without	Thank you for raising this important clinical consideration. We agree this warrants clarification and discussion. While all participants were provided with all four devices, the Goondir VHS model does not prescribe universal use of all devices. Rather, device use is individually tailored based on clinical assessment and chronic	Please refer to the Discussion section.<br><br>Page 16, lines: 7-28

	respiratory symptoms or problems and could result in significant unintended health service burden if there are false positives (common due to factors such as nail polish). Further, there is an ethical dilemma in monitoring people for conditions they don't have, adding to my reservations about monitoring everyone with all four machines in the same way, regardless of degree of multimorbidity. It is also more resource intensive to do so, where these resources could be better targeted and spread across more people. For example, if someone had type 2 diabetes and hypertension, the BP and BSL monitors would be appropriate, but perhaps not scales and oximeter. If someone had severe COPD and was cachectic and a CO2 retainer, then perhaps the oximeter and weight scales would be appropriate, but not BP and BSL monitor. There is also pain / discomfort associated with monitoring BSLs / BP when not required, so this should be addressed in the discussion. We don't wish to cause discomfort to people unless it is advisable on the balance of risk/benefit for their health.	condition profile, guided by health coaches in consultation with clinicians.<br>Our study operationalised "active VHS user" as use of any device (not necessarily all devices) in the past two weeks, recognising that clinically appropriate engagement varies by individual need. However, we did not assess whether device use aligned with clinical indications, which represents an important limitation.<br>We have added discussion of clinical appropriateness considerations and the need for future research examining whether engagement translates to clinically meaningful outcomes.<br><br>**After revision in the Discussion section, it is now written as:**<br>An important consideration is the clinical appropriateness of device use relative to individual chronic condition profiles. While the Goondir VHS model provides all participants with four monitoring devices, device selection is individually tailored based on clinical assessment, with health coaches guiding participants to use only clinically appropriate devices (e.g., participants without diabetes would not monitor blood glucose). However, our study did not assess whether device use aligned with clinical indications. Non-indicated monitoring may pose risks including false positive alerts, increased clinical workload, patient anxiety, and discomfort from unnecessary testing.<br><br>The clinical utility and monitoring purpose vary substantially by device and condition. Blood pressure and blood glucose monitoring in stable chronic disease primarily serve ongoing disease management rather than acute deterioration detection, as symptomatic acute changes would typically prompt direct healthcare contact. Weight monitoring may provide clinical value for detecting fluid retention in heart failure, liver disease, or renal failure, but has limited utility for other conditions. Pulse oximetry is most valuable for respiratory conditions where early desaturation detection may prevent acute decompensation, but routine monitoring without respiratory disease lacks clear clinical benefit.<br><br>Nevertheless, the extremely high multimorbidity burden (92% had multiple conditions) means most participants have clinical indications for multiple	Please also refer to the 'Strengths and Limitations' sub-section of the Discussion section.<br><br>Page 17, lines: 19-57<br><br>& Page 18, lines: 7-12

		devices, and logistical challenges in geographically dispersed populations may make comprehensive device provision more practical than multiple condition-specific deliveries. Future research should examine whether device use aligns with clinical indications, whether engagement improves clinical outcomes, and the comparative effectiveness of universal versus targeted device provision.<br><br>**In the limitation, it is now revised as:**<br><br>We did not assess whether device use aligned with clinical indications for individual participants' chronic conditions, limiting our ability to evaluate the clinical appropriateness of monitoring patterns observed.	
19.	Those with higher use were in medium towns; the opposite of what might be hoped (more remote have less access to health care). This is discussed well.	Thank you.	N/A
20.	It is not quite clear whether the monitoring is for acute or subacute purposes. For example, BSL, BP and 02 may be useful to detect acute deterioration, however clients are likely to be symptomatic (excepting high BP until extreme) which would trigger assessment anyway. Weight scales may only be beneficial in heart/liver/kidney failure population to detect fluid retention acute/subacutely, but may have limited benefit in other populations. A nuanced discussion of how targeted monitoring might be more effective, rather than a blanketed	Thank you for this important observation. We agree this warrants discussion. The Goondir VHS model employs device monitoring primarily for chronic disease management and early detection of gradual deterioration rather than acute emergency detection. We have integrated discussion of monitoring purpose and condition-specific clinical utility into our discussion section.<br><br>Please reference to our response for point #18 above	N/A

	approach, would assist the manuscript.		
21.	Page 15 line 19. While I would be guided by the Indigenous co-authors and governance group, I'm not sure that multi-device platforms is akin to "Indigenous holistic health perspectives," which may be less guided by specific health conditions alone, but rather in concert with connection to inner spirit, community and Country (see Good Spirit Good Life literature). Consider phrasing this differently.	After discussion with our Indigenous project members, we have rephrased the sentence to avoid inappropriately conflating multi-device monitoring with Indigenous holistic health perspectives.<br><br>**After revision in the 'Implications and future research directions' sub-section of the Discussion section, it is now written as:**<br><br>The substantially higher engagement among participants with multiple chronic conditions validates the program's integrated approach, suggesting VHS programs may benefit from prioritising multimorbid individuals with comprehensive monitoring needs.	Please refer to the 'Implications and future research directions' sub-section of the Discussion section.<br><br>Page 18, lines: 25-31
22.	I don't think the described implications for policy and practice are justified based on the findings of this cross-sectional study. This is a usage-based study, not outcome. To roll this out further, at significant cost to clients (through time spent) and ACCHOs, there would need to be a longitudinal outcomes-based assessment. Does this change / improve health and social/emotional wellbeing outcomes? This question cannot be answered by the cross-sectional design in this study.	We agree with this important critique. We have substantially revised the Implications section to reframe findings as preliminary engagement evidence rather than implementation recommendations.<br><br>**After revision in the 'Implications and future research directions' sub-section of the Discussion section, it is now written as:**<br><br>Several research priorities emerge from these findings. The substantially higher engagement among participants with multiple chronic conditions validates the program's integrated approach, suggesting VHS programs may benefit from prioritising multimorbid individuals with comprehensive monitoring needs. However, engagement findings alone cannot justify program expansion. Longitudinal outcome evaluation is essential to determine whether VHS monitoring translates to improved disease control, reduced acute care utilisation, and enhanced quality of life and social-emotional wellbeing outcomes. Economic evaluation weighing implementation costs, including client time investment and ACCHO resource requirements, against potential health benefits is critical before recommending broader rollout.	Please refer to the 'Implications and future research directions' sub-section of the Discussion section.<br><br>Page 18, lines: 25-57

		Understanding barriers to engagement among underrepresented groups warrants investigation. The substantially lower engagement among older adults and those in more remote communities suggests potential barriers requiring further research. Whether addressing these barriers improves health outcomes remains an empirical question. The role of Indigenous health coaches in facilitating engagement requires systematic evaluation. Understanding how culturally grounded support influences outcomes represents a priority research area. This study was conducted within an established ACCHO with strong community trust and governance structures. Generalisability to other settings, particularly mainstream services, cannot be assumed without research examining implementation factors and outcomes across diverse contexts.<br><br>These findings demonstrate feasibility and substantial engagement within a culturally appropriate model, representing an important first step in building an evidence base for VHS in Indigenous primary care. However, outcomes research is required to determine whether engagement translates to meaningful health improvements at acceptable costs before broader implementation can be recommended.	
	**Conclusion**		
23.	The conclusion repeats the findings in the first para of the discussion – it could be made more succinct.	We have substantially shortened and rewritten the Conclusion to avoid repetition of detailed findings already presented in the Discussion, focusing instead on the study's contribution and research implications.	Please refer to the 'Conclusion' section<br><br>Page 19, lines: 15-36
24.	I don't believe the conclusions made are supported by this study. Asserting expanding workforce capacity and roll out on the basis of an engagement study does not yet demonstrate benefit; it's promising, but further longitudinal, outcomes based study is required. Otherwise there is a risk that large	We appreciate your suggestion. We have completely rewritten the Conclusion to appropriately frame findings as preliminary evidence demonstrating feasibility and engagement, while explicitly stating that outcomes research and economic evaluation are required before broader implementation can be recommended.<br><br>**After revision in the Conclusion, it is now written as:**<br><br>This study provides the first comprehensive examination of VHS engagement within	Please refer to the 'Conclusion' section<br><br>Page 19, lines: 15-36

	human and financial resources are used on something that has high engagement, but doesn't improve outcomes. Therefore, I think the conclusions need to reflect the study findings and point towards further study including cost/benefit analysis before considering wider rollout.	Indigenous-led primary care in dispersed rural communities, establishing that digital health monitoring is feasible and can achieve meaningful uptake when delivered through trusted ACCHO settings with culturally appropriate support. By identifying multimorbidity, younger age, and residence in less remote areas as critical engagement determinants, these findings offer important insights for designing equitable digital health interventions in Indigenous communities. However, feasibility and engagement do not establish effectiveness. Without longitudinal clinical outcome data, questions about whether VHS monitoring improves disease management, reduces hospitalisations, or enhances wellbeing remain unanswered. Before broader implementation, outcomes-focused research and economic evaluation are essential to determine whether the observed engagement translates to health improvements justifying the investment required from both clients and health services. This work establishes a foundation for such investigations while identifying priority areas for future research.	

Reference

1. Peduzzi P, Concato J, Kemper E, Holford TR, Feinstein AR. A simulation study of the number of events per variable in logistic regression analysis. J Clin Epidemiol. 1996;49: 1373–1379. doi:10.1016/S0895-4356(96)00236-3

2. Vittinghoff E, McCulloch CE. Relaxing the Rule of Ten Events per Variable in Logistic and Cox Regression. Am J Epidemiol. 2007;165: 710–718. doi:10.1093/aje/kwk052

3. Ali D, Gillie M, Carstarphen KJ. Bridging the digital divide for vulnerable patient telehealth. Innov Aging. 2020;4: 928–928. doi:10.1093/geroni/igaa057.3404

4. Fitzpatrick KM, Ody M, Goveas D, Montesanti S, Campbell P, MacDonald K, et al. Understanding virtual primary healthcare with Indigenous populations: a rapid evidence review. BMC Health Serv Res. 2023;23: 303. doi:10.1186/s12913-023-09299-6

5. Pires G, Lopes A, Correia P, Almeida L, Oliveira L, Panda R, et al. Usability of a telehealth solution based on TV interaction for the elderly: the VITASENIOR-MT case study. Univers Access Inf Soc. 2023;22: 525–536. doi:10.1007/s10209-021-00859-3

6. Khanassov V, Ruiz AS, Rojas-Rozo L, Ilali M, Sourial R. Telemedicine in primary care of older adults: Results of a multi-phase study. Research Square. 2022. doi:10.21203/rs.3.rs-2166856/v1

7. Mathew S, Fitts MS, Liddle Z, Bourke L, Campbell N, Murakami-Gold L, et al. Telehealth in remote Australia: a supplementary tool or an alternative model of care replacing face-to-face consultations? BMC Health Serv Res. 2023;23: 341. doi:10.1186/s12913-023-09265-2

8. Labadorf J, Nichols M, Williams T, Cunanan C, D'Anza B. A Smartphone Is Not Enough: Telehealth Attendance and the Digital Divide. Health Care Science. 2025;4: 259–268. doi:10.1002/hcs2.70033

9. Reeves JJ, Ayers JW, Longhurst CA. Telehealth in the COVID-19 Era: A Balancing Act to Avoid Harm. J Med Internet Res. 2021;23: e24785. doi:10.2196/24785

10. Khoshrounejad F, Hamednia M, Mehrjerd A, Pichaghsaz S, Jamalirad H, Sargolzaei M, et al. Telehealth-Based Services During the COVID-19 Pandemic: A Systematic Review of Features and Challenges. Front Public Health. 2021;9. doi:10.3389/fpubh.2021.711762

11. Seifert A, Batsis JA, Smith AC. Telemedicine in Long-Term Care Facilities During and Beyond COVID-19: Challenges Caused by the Digital Divide. Front Public Health. 2020;8. doi:10.3389/fpubh.2020.601595

12. Australian Bureau of Statistics. Income and work: Census. In: ABS [Internet]. 2021 [cited 20 Apr 2026]. Available: https://www.abs.gov.au/statistics/labour/earnings-and-working-conditions/income-and-work-census/2021

13. Buawangpong N, Pinyopornpanish K, Pliannuom S, Nantsupawat N, Wiwatkunupakarn N, Angkurawaranon C, et al. Designing Telemedicine for Older Adults With Multimorbidity: Content Analysis Study. JMIR Aging. 2024;7: e52031. doi:10.2196/52031

14. Kraef C, van der Meirschen M, Free C. Digital telemedicine interventions for patients with multimorbidity: a systematic review and meta-analysis. BMJ Open. 2020;10: e036904. doi:10.1136/bmjopen-2020-036904

VERSION 2 - REVIEW

Reviewer 2

Name Zanker, Jesse

Affiliation University of Melbourne, Department of Medicine - Royal Melbourne Hospital

Date 05-May-2026

COI

The authors have addressed each point in detail. The interpretations made from the study now match what the study was designed to test. I commend the authors on their thorough and systematic revision of the manuscript.
